# Safety and technical efficacy of early minimally invasive endoscopy-guided surgery for intracerebral haemorrhage: the Dutch Intracerebral haemorrhage Surgery Trial pilot study

**DOI:** 10.1007/s00701-023-05599-2

**Published:** 2023-04-27

**Authors:** Lotte Sondag, Floris H.B.M. Schreuder, Sjoert A.H. Pegge, Jonathan M. Coutinho, Diederik W.J. Dippel, Paula M. Janssen, W. Peter Vandertop, Hieronymus D. Boogaarts, Ruben Dammers, Catharina J.M. Klijn, On behalf of the Dutch ICH Surgery Trial Study Group, part of the CONTRAST consortium, Lotte Sondag, Floris H.B.M. Schreuder, Jelis Boiten, Paul J.A.M. Brouwers, Jonathan Coutinho, M. Heleen den Hertog, Paula M. Janssen, Wilmar M.T. Jolink, L. Jaap Kappelle, Kuan H. Kho, Radboud W. Koot, Paul L.M. de Kort, Wouter A. Moojen, Dharmin Nanda, Onno P.M. Teernstra, Bram van der Pol, Inger R. de Ridder, Marieke J.H. Wermer, Albert van der Zwan, W. Peter Vandertop, Hieronymus D. Boogaarts, Ruben Dammers, Catharina J.M. Klijn, Dana Holl, Anil Can

**Affiliations:** 1 Department of Neurology, Donders Institute for Brain, Cognition and Behaviour, Radboud University Medical Center, Geert Grooteplein Zuid 10, PO-box 9101, 6500HB Nijmegen, The Netherlands; 2 Department of Medical Imaging, Radboud University Medical Center, Nijmegen, The Netherlands; 3 Department of Neurology, Amsterdam University Medical Centers, Academic Medical Center, Amsterdam, The Netherlands; 4 Department of Neurology, Erasmus MC University Medical Center Rotterdam, Rotterdam, The Netherlands; 5 Amsterdam UMC, University of Amsterdam, Department of Neurosurgery, Amsterdam Neurosciences, Neurovascular Disorders, Meibergdreef 9, Amsterdam, The Netherlands; 6 Amsterdam UMC, Vrije Universiteit Amsterdam, Department of Neurosurgery, Amsterdam Neurosciences, Neurovascular Disorders, De Boelelaan, 1117 Amsterdam, Netherlands; 7 Department of Neurosurgery, Radboud University Medical Center, Nijmegen, The Netherlands; 8 Department of Neurosurgery, Erasmus MC University Medical Center, Rotterdam, The Netherlands

**Keywords:** Intracerebral haemorrhage, Neurosurgery, Cerebrovascular disease, Vascular surgery

## Abstract

**Background:**

Previous randomised controlled trials could not demonstrate that surgical evacuation of intracerebral haemorrhage (ICH) improves functional outcome. Increasing evidence suggests that minimally invasive surgery may be beneficial, in particular when performed early after symptom onset. The aim of this study was to investigate safety and technical efficacy of early minimally invasive endoscopy-guided surgery in patients with spontaneous supratentorial ICH.

**Methods:**

The Dutch Intracerebral Haemorrhage Surgery Trial pilot study was a prospective intervention study with blinded outcome assessment in three neurosurgical centres in the Netherlands. We included adult patients with spontaneous supratentorial ICH ≥10mL and National Institute of Health Stroke Scale (NIHSS) score ≥2 for minimally invasive endoscopy-guided surgery within 8 h after symptom onset in addition to medical management. Primary safety outcome was death or increase in NIHSS ≥4 points at 24 h. Secondary safety outcomes were procedure-related serious adverse events (SAEs) within 7 days and death within 30 days. Primary technical efficacy outcome was ICH volume reduction (%) at 24 h.

**Results:**

We included 40 patients (median age 61 years; IQR 51–67; 28 men). Median baseline NIHSS was 19.5 (IQR 13.3–22.0) and median ICH volume 47.7mL (IQR 29.4–72.0). Six patients had a primary safety outcome, of whom two already deteriorated before surgery and one died within 24 h. Sixteen other SAEs were reported within 7 days in 11 patients (of whom two patients that already had a primary safety outcome), none device related. In total, four (10%) patients died within 30 days. Median ICH volume reduction at 24 h was 78% (IQR 50–89) and median postoperative ICH volume 10.5mL (IQR 5.1–23.8).

**Conclusions:**

Minimally invasive endoscopy-guided surgery within 8 h after symptom onset for supratentorial ICH appears to be safe and can effectively reduce ICH volume. Randomised controlled trials are needed to determine whether this intervention also improves functional outcome.

**Trial registration:**

Clinicaltrials.gov: NCT03608423, August 1st, 2018.

**Supplementary Information:**

The online version contains supplementary material available at 10.1007/s00701-023-05599-2.

## Introduction

Spontaneous intracerebral haemorrhage (ICH) accounts for 16–19% of all strokes in the Western world and for 28–32% in low- and middle-income countries [12, 24]. ICH contributes profoundly to stroke-related disability and 30-day case fatality is around 40% [38, 48]. Survival and functional outcome after spontaneous ICH are independently predicted by age, Glasgow Coma Scale (GCS) score, haematoma volume, the presence of intraventricular haemorrhage (IVH) [38], and hematoma growth [9, 40]. Around 20% of patients with ICH show growth of hematoma, mainly within the first 3 h after symptom onset [3]. Neurotoxicity, caused by blood degradation products and plasma-derived components such as thrombin, and inflammation, may lead to the development of perihematomal oedema (PHO). This process starts already within 3 to 4 h after symptom onset [4, 20]. Reduction of the hematoma volume in an early stage may not only alleviate the direct mass effect of the ICH, but may also prevent hematoma growth and development of PHO [35, 51]. Theoretically, this could result in better functional outcome [2, 35]. Increasing evidence including recent narrative [8, 14] and systematic reviews [43, 44] of randomised controlled trials (RCTs) suggests that surgical hematoma evacuation may reduce mortality and improve functional outcome [13, 43, 44, 55]. However, large high-quality RCTs have not established this presumed beneficial effect of surgery [15, 16, 30, 31, 44]. Besides evacuation by means of a craniotomy, several minimally invasive techniques are available to remove the hematoma [2, 17, 36, 41, 55]. Recent case series suggest that endoscopy-guided minimally invasive evacuation by means of aspiration is promising and safe and effectively reduces haemorrhage volume, with few complications [1, 11, 19, 23, 27, 41, 45, 46, 53]. The MISTIE III trial recently failed to demonstrate a beneficial effect on functional outcome of minimally invasive surgery with local application of alteplase followed by hematoma aspiration up to 72 h [16]. The relatively long median time from symptom onset to surgery in MISTIE III (59 h) [16] and in the other large, multicentre, high-quality studies of conventional surgery STICH (30 h) [30] and STICH-II (26 h) [31] might in part explain the lack of a treatment effect. An individual patient data meta-analysis of RCTs found that outcome improved after surgery compared to standard medical management, if surgery was performed within 8 h [13]. However, one study showed that ultra-early surgery (within 4 h) was associated with rebleeding [34], whilst other studies have shown that ultra-early surgery (<4–6 h) is safe [25, 26]. In addition, a recent study showed that for every hour that patients were operated earlier, there was a 5% increase in the odds of good functional outcome at 6 months [22].

Well-designed RCTs are needed to provide definitive and guideline changing evidence whether or not early minimally invasive and endoscopy-guided surgical evacuation would reduce mortality after sICH and improves functional outcome [43]. As a prelude to such a large randomised clinical trial, the aim of the Dutch ICH Surgery Trial (DIST) pilot study was to investigate safety and technical efficacy of minimally invasive endoscopy-guided surgery for the treatment of spontaneous supratentorial ICH within 8h after symptom onset.

## Methods

### Study design

The DIST pilot study was a multicentre, prospective, non-randomised intervention study in the Netherlands, with blinded endpoint assessment, investigating the safety and technical efficacy of minimally invasive endoscopy-guided surgical evacuation of supratentorial spontaneous ICH within 8h after symptom onset, in addition to standard medical management (Clinicaltrials.gov: NCT03608423; http://dutch-ich.nl). Patients underwent minimally invasive endoscopy-guided surgery in addition to standard medical management in three hospitals. In seven other centres, patients received standard medical management only.

We aimed to include 40 patients in the surgical arm, with a minimum of 10 patients per centre, to be able to obtain clinically meaningful data on the safety and technical efficacy of our intervention, assuming we could show that on average we would be able to reduce ICH volume with at least 60%.

Here, we report the results of the 40 patients who underwent surgery. This study was approved by the medical ethics committee of the Erasmus MC University Medical Centre Rotterdam (MEC_2018-012).

### Participants

Patients were eligible if they met the following inclusion criteria: age ≥18 years, supratentorial spontaneous (non-traumatic) ICH ≥10 mL confirmed by non-contrast computed tomography (NCCT) scan, National Institutes of Health Stroke Scale (NIHSS) score ≥2, and initiation of surgery possible within 8 h after symptom onset. Written informed consent was obtained from all patients or their legal representatives. The diagnosis of spontaneous ICH was based on the absence of a causative macrovascular lesion (i.e. aneurysm, arteriovenous malformation, dural arteriovenous fistula, cerebral venous sinus thrombosis) on computed tomography angiography (CTA) scan performed immediately following the NCCT [18, 49].

ICH volumes in the acute setting were calculated using the ABC/2 method. We verified these volumes if they were close to the limit of 10mL using volume measurements in Brainlab® (AG, München, Germany). Screening logs were not kept. Patients were excluded from participation if they had any other known cause of the ICH (i.e. tumour, cavernoma), pre-stroke disability that interfered with the assessment of functional outcome (i.e. pre-stroke modified Rankin Scale (mRS) score >2) [6], untreated coagulation abnormalities, pregnancy, or when a patient was moribund (i.e. coning, dilated unresponsive pupils). Patients who were using a vitamin K antagonist could be included after reversal of the anticoagulation effect (INR ≤1.3). Patients who were using direct oral anticoagulants were excluded. The use of antiplatelet therapy, including clopidogrel, was not an exclusion criterion.

At baseline, we collected data on age and sex, medical history (presence of hypertension, atrial fibrillation, diabetes mellitus, hypercholesterolemia, previous ischemic or haemorrhagic stroke, transient ischemic attack, previous major bleeding, myocardial infarction, premorbid cognitive dysfunction, premorbid disability on the mRS), and the use of medication (antiplatelet therapy, antihypertensive agents, oral anticoagulants, statins). In addition, we collected systolic and diastolic blood pressure, GCS score, NIHSS score, and ICH-Grading Scale (ICH-GS, with higher scores indicating worse prognosis) score [42] at baseline. An independent neuroradiologist assessed baseline NCCTs for ICH volume, ICH location, presence of intraventricular, subdural or subarachnoid extension, PHO volume, hydrocephalus, and CTA for presence of a spot sign, blinded to clinical characteristics and to safety and technical efficacy outcomes. The same independent and blinded neuroradiologist assessed the NCCT that was performed at 24 h. We calculated time intervals from symptom onset (or last seen well) to NCCT. All patients underwent assessment of the NIHSS at 24 h and 7 days or discharge. All SAEs within 30 days were reported.

### Intervention

Neurosurgeons with experience in endoscopic neurosurgery for other purposes were trained to perform the surgical procedures. They followed a prespecified protocol (Online resource data) and performed surgery under general anaesthesia. The baseline NCCT was uploaded into neuronavigational software (Brainlab® AG, München, Germany). Neurosurgeons selected a trajectory for evacuation that allowed safe access to the longest possible axis of the haemorrhage, in a similar way as in the ICES study [50], and described earlier as the stereotactic intracerebral haemorrhage underwater blood aspiration (SCUBA) and ADAPT technique [21, 41]. The image-guidance probe was positioned over the candidate entry point to assess whether or not the endoscope sheath would transgress any critical functional areas. Neurosurgeons created a burr hole of 1.5–2.0 cm, opened the dura, and incised and coagulated the cortical surface. A localisation array was attached to the selected neuro-endoscopic sheath and registered to the navigation system. The sheath was stabilised into the target zone, typically at 2/3 of the length of the long axis, using neuronavigation. The neuro-endoscope was inserted into the sheath and under direct visualisation the Artemis^TM^ Neuro Evacuation Device (Penumbra Inc. Alameda, California, USA) was placed through the working channel of the endoscope. Intermittent irrigation through the endoscope and hematoma aspiration via the Artemis^TM^ device was performed under direct visualisation until a clear working view was created, revealing the hematoma cavity. This was repeated until no further clot could be evacuated at this location. In case of active bleeding, irrigation was continued until the bleeding stopped. Once haemostasis was obtained, the endoscope sheath was retracted to a point approximately 1/3 of the hematoma cavity. The suctioning and irrigation process was then repeated at this point. Suctioning was continued until the surgeon estimated to have removed at least 75–80% of the hematoma volume. Then, the endoscope was introduced for a final inspection of the cavity to check for any ongoing bleeding that required additional treatment through irrigation and occasional bipolar coagulation. The dura and skin were closed. An NCCT was performed intra-operatively or immediately after evacuation to confirm adequate haemorrhage evacuation. Whether or not to opt for resumption of the surgery to evacuate any residual haemorrhage was at the surgeons’ discretion. Patients were admitted to the neuro-intensive care unit or a dedicated stroke unit, and were treated according to the Dutch national stroke guideline [10]. We recorded time from symptom onset to arrival in the operating room, to first incision and to skin closure, blood pressure measurements during surgery, the type of endoscope that was used, the occurrence of active bleeding or rebleeding during surgery, conversion to craniotomy, and whether the surgery was performed in a hybrid operating room.

### Outcomes

The primary safety outcome was death within 24 h or neurological deterioration with an increase of ≥4 points on the NIHSS at 24 h. Primary safety outcomes were adjudicated by an independent committee, consisting of two neurologists and one neurosurgeon. Secondary safety outcomes were ICH volume at 24 h, death at 7 and 30 days, and 7-day procedure-related complications. Additionally, we recorded all SAEs within 30 days. The primary technical efficacy outcome was the percentage of volume reduction comparing the ICH volumes on the NCCT at baseline and the NCCT at 24 h. All images were centrally assessed by an independent neuroradiologist, blinded for baseline characteristics and safety and technical efficacy outcomes. Secondary technical efficacy outcomes were the percentage of patients with a clot volume reduction of ≥60%, the percentage of patients with a clot volume reduction ≥80%, the percentage of patients with a remaining clot volume ≤15mL at 24 h, and the number of patients in whom the minimally invasive procedure was converted to craniotomy. (Re)bleed was defined as a higher ICH volume at 24 h than at baseline or postoperatively (irrespective of change in clinical symptoms). The Data Safety Monitoring Board (DSMB) would consider to advise to hold the study when the lower limit of the 95% CI of the proportion of patients with the occurrence of the primary safety outcome reached 40%.

### Data analyses

Baseline characteristics are displayed as number of patients with corresponding percentages, mean with standard deviation (SD), or median with 25% and 75% interquartile range (IQR) as appropriate. ICH volume at baseline and 24 h NCCT, and IVH volume and PHO volume at baseline NCCT, were measured via manual segmentation using ITK-SNAP 3.8 (http://www.itksnap.org) [54] and volumes were calculated using MATLAB 2018a, based on the number of voxels and voxel size in three directions. For safety outcomes, we calculated absolute numbers and percentages with 95% confidence intervals (CIs). An independent DSMB monitored the study with the aim to advise to stop the study if the lower limit of the 95% CI of the percentage of patients with a primary safety outcome would reach 40%. Reduction of hematoma volume was expressed as median (IQR) percentage volume reduction. To obtain insight into the effect of a potential learning curve on technical efficacy, we assessed the first and second half of patients separately as an exploratory analysis. We split the group by the first half and second half within each centre. In case of unequal numbers, the second group would be the largest.

## Results

**Fig. 1:**
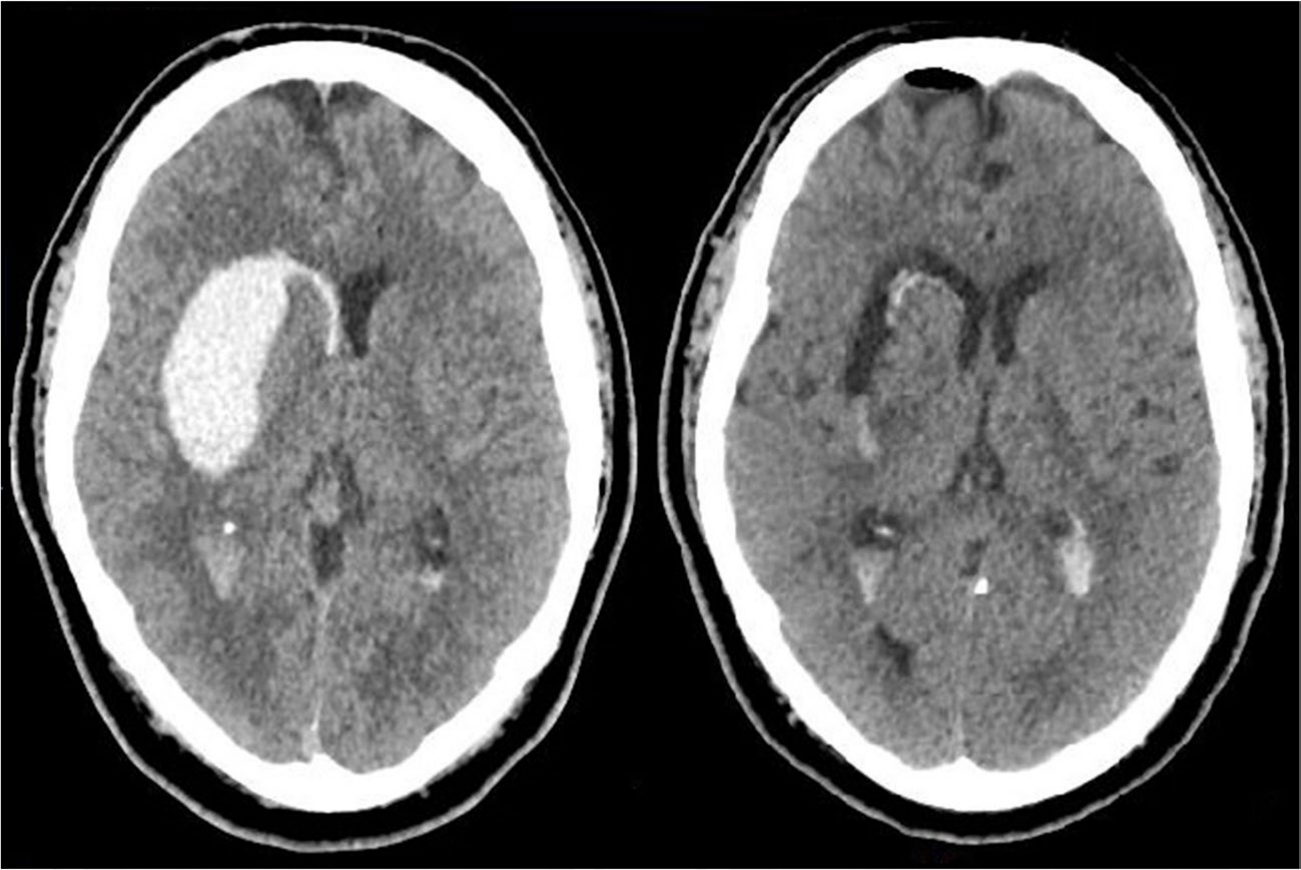
A 56-year-old man with a history of hypertension presented to the emergency room with a GCS score of E3M6V3, an NIHSS score of 21 and ICH-GS score of 9. NCCT showed a non-lobar haemorrhage on the right of 42.9 mL, with ventricular extension. CTA showed no spot sign. Surgery was started 7 h and 40 min after symptom onset. Postoperatively, the remaining volume was 3.0 mL, a reduction of 93.1%

Forty patients were included between March 2019 and January 2021. We experienced delays at the start due to limited availability of the surgical devices, and during the study because of the COVID-19 pandemic with temporary halting of clinical research activities and limited availability of operation room capacity and ICU beds. Demographics and baseline characteristics are summarised in Table 1. Median ICH volume at baseline was 47.7mL (IQR 29.4–72.0mL; 2 patients had a baseline ICH volume <15mL). Median time from symptom onset to the start of the surgery (first incision) was 6 h and 43 min (IQR 5 h 26 min–7 h 56 min). Median duration of the procedure (first incision to skin closure) was 68 min (IQR 55–106 min). Median ICH volume after 24 h was 10.5mL (IQR 5.1–23.8mL). Further details of the surgical procedures are summarised in Table 2. Online resource table 2 shows the baseline characteristics and technical outcome parameters according to baseline ICH volume subgroups (<30mL and ≥30mL). An illustrative case is shown in Fig. 1. Twelve patients had a CTA spot sign, of whom five had active bleeding during surgery. Of a total of 19 patients with active bleeding during surgery (Table 2), 14 did not have a spot sign at baseline.

**Table 1:** Patient characteristics

Characteristic	*N*=40
Age, mean (SD)	59.5 (13.6)
Men, *n* (%)	28 (70.0)
Medical history of	
Hypertension, *n* (%)	14 (35.0)
Atrial fibrillation, *n* (%)	4 (10.0)
Diabetes mellitus, *n* (%)	6 (15.0)
Hypercholesterolemia, *n* (%)	9 (22.5)
Ischemic stroke, *n* (%)	1 (2.5)
TIA, *n* (%)	5 (12.5)
Previous ICH, *n* (%)	2 (5.0)
Myocardial infarction, *n* (%)	3 (7.5)
Premorbid cognitive dysfunction, *n* (%)	1 (2.5)
Medication at baseline, use of	
Antiplatelet therapy, *n* (%)	11 (27.5)
Acetylsalicylic acid	7 (17.5)
Clopidogrel	4 (10.0)
Antihypertensive drug(s), *n* (%)	11 (27.5)
Vitamin K antagonist, *n* (%)	1 (2.5)
Statin, *n* (%)	13 (32.5)
Pre-ICH mRS score 0, *n* (%)	33 (82.5)
Pre-ICH mRS score 1, *n* (%)	5 (12.5)
Pre-ICH mRS score 2, *n* (%)	2 (5.0)
Time from symptom onset or last seen well to arrival study centre, median (IQR)	3h 32min (1h 49min–4h 52min)
Glasgow Coma Scale score (on admission), median (IQR)	12 (9–14)
GCS score <9, *n* (%)	5 (12.5)
GCS score 9–12, *n* (%)	17 (42.5)
GCS score 13–15, *n* (%)	18 (45)
NIHSS score (on admission), median (IQR)	19 (13–22)
Systolic blood pressure, mmHg (on admission), mean (SD)	174 (31)
Diastolic blood pressure, mmHg (on admission), mean (SD)	102 (28)
Baseline NCCT scan	
ICH volume (mL), mean (SD)	50 (23)
Lobar location, *n* (%)	11 (27.5)
Left hemispheric location, n (%)	21 (52.5)
IVH present, *n* (%)	19 (47.5)
IVH volume (mL), median (IQR)	0 (0-17)
Hydrocephalus, *n* (%)	11 (27.5)
Perihematomal oedema volume (mL), median (IQR)^a^	14 (7-29)
Subarachnoid extension of ICH, *n* (%)	9 (22.5)
Finger like projections, *n* (%)	9 (22.5)
Time from onset symptoms (or last seen well) to baseline NCCT, median (IQR)	3h 46min (1h 30min–5h 33min)
CT-angiography spot sign present, *n* (%)^b^	12 (30)
ICH-GS score at inclusion, *n* (%)	
ICH-GS-score 5	1 (2.5)
ICH-GS-score 6	3 (7.5)
ICH-GS-score 7	9 (22.5)
ICH-GS-score 8	10 (25.0)
ICH-GS-score 9	11 (27.5)
ICH-GS-score 10	4 (10.0)
ICH-GS-score 11	2 (5.0)

*CT* computed tomography scan, *GCS* Glasgow Coma Scale, *ICH* intracerebral haemorrhage, *ICH-GS* intracerebral haemorrhage grading scale, *IQR* interquartile range, *IVH* intraventricular haemorrhage (extension), *mRS* modified Rankin Scale, *NIHSS* National Institutes of Health Stroke Scale, *TIA* transient ischemic attack

^a^Two patients had no perihematomal oedema

^b^No CTA available in 2 patients

**Table 2:** Surgical treatment

	*N*=40
Time from symptom onset to arrival operating room (hours, min), median (IQR)	5h 53 (4h 15–7h 14)
Interval from symptom onset or last seen well to first cut <4 hours, *n* (%)	3 (7.5)
Interval from symptom onset or last seen well to first cut <6 hours, *n* (%)	21 (52.5)
Surgery performed on hybrid OR, *n* (%)	8 (20.0)
Endoscope used, *n* (%)	
Lotta (Storz)	39 (97.5)
Minop (B.Braun)	1 (2.5)
Active bleeding during surgery, *n* (%)	19 (47.5)
Rebleeding or new intracranial bleeding during surgery, *n* (%)	3 (7.5)
Highest systolic blood pressure during surgery (mmHg), median (IQR)	162 (139–189)
Lowest systolic blood pressure during surgery (mmHg), median (IQR)	117 (97–140)
Re-operation after intra-operative or direct post-operative NCCT, *n* (%)^a^	8 (20.0)
EVD placement, *n* (%)^b^	9 (22%)

*IQR* interquartile range, *OR* operation room

^a^In these patients, median duration of surgery (from first cut to skin closure after resumption of operation) was 2 h and 58 min

^b^EVD was placed either during the initial surgical procedure, or in a later stadium during admission

### Primary safety outcomes

Of the 40 included patients, six (15%; 95% CI 5.7–29.9%) had a primary safety outcome. One patient died within 24 h (2.5%; 95% CI: 0.1–13.2%) and five patients (12.5%; 95% CI: 4.2–26.8%) experienced an increase in NIHSS score of ≥4 points at 24 h, compared to baseline. Two of these five patients deteriorated in the emergency room before surgery had commenced. Two other patients of these five patients remained intubated at 24 h (but not at baseline), which increased their NIHSS scores. The fifth patient had disorientation in time and an increase in the severity of a left-sided paresis at 24 h, which partly recovered over time. Further clinical details of the primary outcomes are outlined in Online resource Table 3.

### Technical efficacy

The median percentage volume reduction on CT at 24 h was 78% (IQR 50–89%) compared to baseline NCCT. Table 3 summarises the secondary safety and technical efficacy outcomes. In the majority of patients, the ICH volume was reduced with 60% or more and the remaining volume ≤15mL. In one patient (2.5%), the surgical procedure was converted to a craniotomy because of continuing active bleeding. Median proportional ICH volume reduction in the first half of patients was 70.5% (IQR 35.5–86.2) and 82.6% (IQR 55.4–89.8) in the second half of patients, whilst case mix appeared largely comparable (Table 4; Online resource Fig).

**Table 3:** Secondary safety and efficacy outcomes

	*N* = 40
ICH volume at 24 h (mL), median (IQR)	10.5 (5.1–23.8)
Secondary safety outcomes, *n* (%)	
Death within 7 days	1 (2.5%)
Death within 30 days	4 (10.0%)
Serious adverse events <7 days, excluding primary safety outcomes	15 (in 11 patients)
Serious adverse events between 8 and 30 days	22 (in 13 patients)
Secondary technical efficacy outcomes, *n* (%)	
Patients with clot volume reduction ≥60%	28 (70.0)
Patients with clot volume reduction ≥80%	19 (47.5)
Patients with remaining clot volume ≤15mL	23 (57.5)
Conversion to craniotomy	1 (2.5)

*ICH* intracerebral haemorrhage, *IQR* interquartile range

**Table 4:** Baseline characteristics and surgical results in first and second half of surgical patients

Baseline characteristics	First half of surgical patients ( *n*=19)	Second half of surgical patients (*n*=21)
Age (years), median (IQR)	57 (45–63)	63 (57.5–69.5)
Men, *n* (%)	13 (68)	15 (71)
ICH volume baseline (mL), median (IQR)	49.7 (29.4–73.7)	42.9 (28.9–65.9)
IVH, *n* (%)	11 (57.9)	8 (38.1)
NIHSS baseline, median (IQR)	20 (19–22)	17 (9.5–20.5)
GCS baseline, median (IQR)	10 (9–13)	13 (10–14.5)
ICH-GS score, median (IQR)	9 (7–9)	8 (7–9)
**Outcomes**		
Time from symptom onset or last seen well to first incision, median (IQR)	6h 35min (5h 19min–8h 10min)	7h 12min (5h 35min–7h 55min)
Postoperative remaining volume (mL), median (IQR)	16.7 (5.0–30.9)	8.4 (4.4–23.0)
Absolute ICH volume reduction (mL), median (IQR)	26.6 (15.5–41.6)	35.9 (19.3–50.1)
ICH volume reduction ≥80%, *n* (%)	6 (31.6)	13 (61.9)
ICH volume reduction ≥60%, *n* (%)	12 (63.2)	16 (76.2)
Postoperative remaining volume ≤15mL	9 (47.4)	14 (66.7)
Duration of the procedure (first incision to skin closure) median min (IQR)	84.0 (52.0–112.0)	62.0 (55.5–89.5)

*GCS* Glasgow Coma Scale score, *ICH* intracerebral haemorrhage, *ICH-GS* intracerebral haemorrhage grading scale, *IQR* interquartile range, *IVH* intraventricular haemorrhage (extension), *NIHSS* National Institutes of Health Stroke Scale

### Secondary safety outcomes

Case fatality at 30 days was 10% (95% CI 2.5–23.7%, *n*=4). Besides the primary safety outcomes, 16 SAEs within the first 7 days were reported in 11 patients, of whom 2 patients that also had a primary safety outcome (Table 5). Another 23 SAEs were registered between 8 and 30 days in 13 patients. Of these 13 patients, 11 patients also had an earlier SAE reported within 7 days, of whom two also had a primary safety outcome. No device-related SAEs were reported. One patient had an intracranial infection within 7 days (at day 4) with a positive cerebrospinal fluid (CSF) culture. None of the 9 patients that were treated with an external ventricular drain (EVD) developed an intracranial infection proven with positive CSF cultures. Three of these patients (7.5%) were pragmatically treated for a suspected intracranial infection, based on clinical symptoms (*n*=2) or increased leukocyte count in the CSF without a decreased glucose level (*n*=1) in addition to clinical symptoms, but CSF cultures were not performed or remained negative.

**Table 5:** Overview of SAEs (primary safety outcomes excluded) per organ system

	SAE ≤7 days	SAE 8–30 days
Gastro-intestinal disorders	**–**	2
Infections and infestations (other than intracranial infection or pneumonia)	1	1
Nervous system disorders		
ICH progression (CT confirmed)	1^a^	2^c^
Clinical deterioration; ICH progression suspected but not CT confirmed	2^b^	1
ICH progression with epidural hematoma	**–**	1^c^
ICH progression with hydrocephalus	**–**	1
Intracranial infection, with positive CSF culture	1^d,e^	**–**
Suspected intracranial infection (without positive CSF culture)	**–**	4^d,e^
Postoperative site infection	**–**	1^d^
Other	**–**	1
Psychiatric disorders	3	1
Renal and urinary disorders	1	1
Respiratory, thoracic and mediastinal disorders		
Pneumonia	5	3
Pulmonary embolism	0	1
Reintubation	2	2
Other	0	1
**Total**	16	23

*ICH* intracerebral haemorrhage

^a^One patient had ICH progression at the day of surgery with less than four points NIHSS increase

^b^Two patients deteriorated clinically but no imaging was performed to confirm ICH progression

^c^All three occurred in the same patient

^d^One patient had an intracranial infection within seven days as well as a postoperative site infection followed by an intracranial infection between eight and 30 days

^e^One patient had an intracranial infection on day four, with a positive cerebrospinal fluid (CSF) culture for *Streptococcus mitis*. This patient was not treated with an EVD. Of the other three patients that had a suspected intracranial infection after 7 days, all have had an EVD and none of them had positive CSF cultures

Four patients (10%) had a rebleed within 30 days, of whom two within 7 days. In two patients, the rebleed occurred on the first day (3.5 and 3 h after surgery that was started 4 h 15 min and 8 h 13 min after symptom onset). In the other two patients (surgery started after 7 h 40 min and 4 h 11 min after symptom onset), a rebleeding occurred at day 8 and 11. In the latter, therapeutic heparin was started because of a pulmonary embolism. All four patients with a rebleed underwent another surgical procedure: in two an EVD was placed, one underwent a hemicraniectomy with hematoma evacuation and another patient was treated with bilateral EVD-placement and craniotomy. In this 63-year-old patient with a 48-mL lobar hematoma, a diagnosis of cerebral amyloid angiopathy was suspected. In one of the patients treated with EVD, uncontrolled postoperative hypertension may have contributed to the ICH progression. A full list of SAEs per organ system can be found in Table 5.

## Discussion

In the DIST pilot study, minimally invasive endoscopy-guided surgery within 8 h of symptom onset in patients with a spontaneous supratentorial ICH appeared safe and could be performed with good technical results. Technical efficacy tended to become better in the second half of the study, suggesting a learning curve for the procedure.

Our study differs from previous studies in that all patients had minimally invasive endoscopy-guided hematoma evacuation within the time window of 8 h after symptom onset. Previous non-randomised studies of endoscopy-guided minimally invasive surgery included patients up to 16 days after symptom onset with a median time to surgery of 19 h to 2.3 days [23, 46, 50]. In these studies, only a minority of patients was operated on within 8 h after symptom onset. The optimal timing of surgical evacuation is still a topic for debate. In a systematic review and meta-regression analysis, we recently showed that surgery seemed to be more effective when performed earlier after symptom onset [44]. Another review of 15 high-quality studies including 2152 patients found no difference in the risk of death or dependence between minimally invasive surgery performed within 24 h (OR 0.49, 95% CI 0.38–0.63, five studies) or within 72 h (OR 0.57, 95% CI 0.43–0.76, 12 studies) as compared with medical care or conventional craniotomy [43]. In both reviews, analyses were based on study level data and not on individual patient data. In addition, only one of the included studies restricted inclusion to patients in whom surgery could be started within 8 h after symptom onset [37]. In an observational study in 59 patients without a spot sign on CTA, the authors found no difference in rebleeding rates after stereotactic aspiration within 6 h or after 6 h after symptom onset [26]. In contrast, a small pilot study that assessed hematoma evacuation by means of a craniotomy within 4 h after symptom onset was stopped early because rebleeding occurred in four of 11 patients within 24 h after surgery [34]. In our study, a rebleed did not occur in the three patients in whom surgery was started within 4 h after symptom onset, and occurred in only one of 21 patients in whom surgery was started within 6 h. In MISTIE III (median time between symptom onset and start of surgery 58 h), six of 255 patients (2%) had a symptomatic bleeding within 72 h, and 81 patients (32%) an asymptomatic bleeding [16]. In the ICES study [50] (median time between symptom onset and start of surgery 30 h), three of 14 patients (21%) had an asymptomatic bleeding within 72 h, defined as an increase of at least 5mL and less than 2 points increase on the GCS motor score. Although a previous cohort study in 143 patients with spontaneous ICH who underwent endoscopic hematoma evacuation found no association between hypertension and postoperative rebleeding [32], we cannot exclude that elevated blood pressure after surgery may have contributed to rebleeding in one of the patients in our study. In practice, we strive for a systolic blood pressure of <160 mmHg. We could not confirm the findings of previous studies that the CTA spot sign is associated with postoperative rebleeding [32, 33]. In addition, we found no association between the presence of a CTA spot sign and intra-operative active bleeding, in contrast to what has previously been suggested by others [33]. In that study, 23% of patients were operated within 8 h, and could also have infratentorial ICH, and more frequently had a coagulopathy [33] than in our study. Based on our results, we suggest that patients with a CTA spot sign should not be excluded from future trials. Our 30-day case fatality of 10% is similar to that of 9–14% found in MISTIE III [16], as well as in ICES [50] (7%, 95% CI 1.8–33.9%) and other prospective studies investigating minimally invasive endoscopy-guided surgery [23, 46], in which patients were operated on at a median of 30 h to 3.2 days after symptom onset [16, 23, 46, 50].

The low percentage of intracranial infection (*n*=1; 2.5%) in this study was similar to that in MISTIE III (*n*=2/255; 1%) [16], ICES (*n*=0/14) [50], and another prospective study investigating minimally invasive endoscopy-guided surgery (*n*=2/49; 4%) [53].

The technical efficacy in our study was similar to the 54 to 97% postoperative ICH volume reduction reported in other studies of various minimally invasive techniques performed at later times after symptom onset (median time between symptom onset to surgery ranging from 19 h to 2.3 days) [23, 28, 46, 47, 50]. Hence, technical efficacy appears no reason to defer surgery to later time-points.

Previous studies on endoscopic surgery have shown that the evacuation rate improves with more experience [5, 28, 50], and that better technical results are associated with improved functional outcomes [16]. In our study, there also appeared to be a learning curve, with a suggestion of slightly better technical results in the second part of the study (albeit with overlapping 95% confidence intervals). In MISTIE -III, a threshold of four patients per surgeon and seven per centre was found, after which no patient had poor end of treatment volumes [16]. This learning curve is important to take into account in the design of future studies. The IDEAL recommendations for the design and reporting of studies on surgical and interventional therapy innovations [29] advise to monitor the quality of the intervention, including pre-operative care, surgery, and post-operative care. For RCTs these recommendations additionally advise to evaluate the learning curve, ideally with Bayesian hierarchical models, and to minimise potential harms by mentoring and training, and to demonstrate that the technique can be widely adopted by surgeons [7, 29]. Because of the small sample size, we refrained from formal analysis of the learning curve.

Based on the results of this study and those of others, we conclude that high-quality RCTs are needed to investigate whether minimally invasive surgery improves functional outcome in patients with spontaneous supratentorial ICH. It remains to be determined whether the timing is indeed a key factor in the success of this treatment [44, 52], but ‘time is brain’ may also hold true for ICH [21, 39]. The existence of a learning curve should be taken into account in the design of such trials.

Several RCTs comparing minimally invasive endoscopy-guided surgery with standard medical management in different time windows are ongoing: MIND (NCT03342664) investigates minimally invasive surgery with the Artemis^TM^ device within 72 h after symptom onset, ENRICH (NCT02880878) investigates minimally invasive surgery with the Brainpath® device by NICO Corporation (Indianapolis, Indiana, USA) within 24 h after symptom onset, and EVACUATE (NCT04434807) is investigating minimally invasive aspiration by the Aurora Surgiscope® by Rebound Therapeutics® (Irvine, California, USA) within 8 h after symptom onset. In the Dutch Intracerebral Haemorrhage Surgery Trial (DIST, NCT05460793), we will investigate whether treatment with minimally invasive endoscopy-guided surgery within 8 hours after symptom onset in addition to medical management improves functional outcome after 6 months in comparison with medical management alone. DIST has started in October 2022 and is estimated to be completed in 2027. Strengths of the DIST pilot study are its prospective, multicentre design and the broad inclusion criteria, without limitations for age, GCS, or the presence of a CTA spot sign, following a standardised surgical protocol. In addition, all imaging to determine technical efficacy was centrally assessed by an independent neuroradiologist, blinded for baseline characteristics and outcomes. Furthermore, we assessed the difference in technical efficacy between the first half and second half of patients, highlighting the importance of training.

Our study also has some limitations. First, the sample size was small. Second, we were not able to report on the effect of early minimally invasive endoscopy-guided surgery on functional outcome. Third, we did not keep screening logs. Furthermore, results might not be generalisable to other countries than the Netherlands. In particular, the distance to a neurosurgical centre in the Netherlands is small in comparison with other countries.

## Conclusion

This study shows that in patients with supratentorial ICH, minimally invasive endoscopy-guided surgery within 8 h after symptom onset appears safe and technically effective. An RCT is needed to assess whether this intervention improves functional outcome.

## Supplementary information

ESM 1 (DOCX 59 kb)

## Abbreviations

CTA: Computed tomography angiography
DSMB: Data Safety Monitoring Board
EVD: External ventricular drain
GCS: Glasgow Coma Scale score
ICH: Intracerebral haemorrhage
ICH-GS: ICH-Grading Scale
IQR: Interquartile range
INR: International normalised ratio
IVH: Intraventricular haemorrhage
mRS: modified Rankin Scale
NIHSS: National Institute of Health Stroke Scale
NCCT: Non-contrast computed tomography
OR: Odds ratio
PHO: Perihematomal oedema
RCT: Randomised controlled trial
SAE: Serious adverse event
TIA: Transient ischemic attack

## Acronyms

DIST: The Dutch Intracerebral Haemorrhage Surgery Trial
ICES: Intraoperative sterotactic Computed tomography-guided Endoscopic Surgery for brain haemorrhage trial
MISTIE: Minimally Invasive Surgery plus rt-PA for Intracerebral haemorrhage Evacuation trial
STICH: Surgical Treatment for Intracerebral Haemorrhage trial

## References

[1] Ali M, Zhang X, Ascanio LC et al (2022) Long-term functional independence after minimally invasive endoscopic intracerebral hemorrhage evacuation. J Neurosurg:1–11 10.3171/2022.3.JNS22286 35561694

[2] Al-Kawaz MN, Hanley DF, Ziai W (2020) Advances in therapeutic approaches for spontaneous intracerebral hemorrhage. Neurotherapeutics. 10.1007/s13311-020-00902-w PMC7851203 32720246

[3] Al-Shahi Salman R, Frantzias J, Lee RJ (2018). Absolute risk and predictors of the growth of acute spontaneous intracerebral haemorrhage: a systematic review and meta-analysis of individual patient data. Lancet Neurol.

[4] Askenase MH, Sansing LH (2016). Stages of the inflammatory response in pathology and tissue repair after intracerebral hemorrhage. Semin Neurol.

[5] Awad IA, Polster SP, Carrion-Penagos J (2019). Surgical performance determines functional outcome benefit in the minimally invasive surgery plus recombinant tissue plasminogen activator for intracerebral hemorrhage evacuation (MISTIE) procedure. Neurosurgery.

[6] Bamford JM, Sandercock PA, Warlow CP, Slattery J (1989). Interobserver agreement for the assessment of handicap in stroke patients. Stroke..

[7] Cook JA, Ramsay CR, Fayers P (2004). Statistical evaluation of learning curve effects in surgical trials. Clin Trials.

[8] Cordonnier C, Demchuk A, Ziai W, Anderson CS (2018). Intracerebral haemorrhage: current approaches to acute management. Lancet..

[9] Davis SM, Broderick J, Hennerici M (2006). Hematoma growth is a determinant of mortality and poor outcome after intracerebral hemorrhage. Neurology..

[10] Dippel DWJ, van der Worp HB, Hofmeijer J (2021). Richtlijn herseninfarct en hersenbloedingen.

[11] Fiorella D, Arthur A, Schafer S (2015). Minimally invasive cone beam CT-guided evacuation of parenchymal and ventricular hemorrhage using the Apollo system: proof of concept in a cadaver model. J Neurointerv Surg.

[12] GBD-2019-Stroke-Collaborators (2021). Global, regional, and national burden of stroke and its risk factors, 1990-2019: a systematic analysis for the Global Burden of Disease Study 2019. Lancet Neurol.

[13] Gregson BA, Broderick JP, Auer LM (2012). Individual patient data subgroup meta-analysis of surgery for spontaneous supratentorial intracerebral hemorrhage. Stroke..

[14] Gross BA, Jankowitz BT, Friedlander RM (2019). Cerebral intraparenchymal hemorrhage: a review. JAMA..

[15] Hanley DF, Thompson RE, Muschelli J (2016). Safety and efficacy of minimally invasive surgery plus alteplase in intracerebral haemorrhage evacuation (MISTIE): a randomised, controlled, open-label, phase 2 trial. Lancet Neurol.

[16] Hanley DF, Thompson RE, Rosenblum M (2019). Efficacy and safety of minimally invasive surgery with thrombolysis in intracerebral haemorrhage evacuation (MISTIE III): a randomised, controlled, open-label, blinded endpoint phase 3 trial. Lancet..

[17] Hersh EH, Gologorsky Y, Chartrain AG, Mocco J, Kellner CP (2018). Minimally invasive surgery for intracerebral hemorrhage. Curr Neurol Neurosci Rep.

[18] Hilkens NA, van Asch CJJ, Werring DJ (2018). Predicting the presence of macrovascular causes in non-traumatic intracerebral haemorrhage: the DIAGRAM prediction score. J Neurol Neurosurg Psychiatry.

[19] Hsu CH, Chou SC, Kuo LT (2022). Minimally invasive neurosurgery for spontaneous intracerebral hemorrhage-10 years of working progress at National Taiwan University Hospital. Front Neurol.

[20] Keep RF, Hua Y, Xi G (2012). Intracerebral haemorrhage: mechanisms of injury and therapeutic targets. Lancet Neurol.

[21] Kellner CP, Chartrain AG, Nistal DA (2018). The Stereotactic Intracerebral Hemorrhage Underwater Blood Aspiration (SCUBA) technique for minimally invasive endoscopic intracerebral hemorrhage evacuation. J Neurointerv Surg.

[22] Kellner CP, Song R, Ali M (2021). Time to evacuation and functional outcome after minimally invasive endoscopic intracerebral hemorrhage evacuation. Stroke..

[23] Kellner CP, Song R, Pan J (2020). Long-term functional outcome following minimally invasive endoscopic intracerebral hemorrhage evacuation. J Neurointerv Surg.

[24] Krishnamurthi RV, Ikeda T, Feigin VL (2020). Global, regional and country-specific burden of ischaemic stroke, intracerebral haemorrhage and subarachnoid haemorrhage: a systematic analysis of the Global Burden of Disease Study 2017. Neuroepidemiology..

[25] Kuo LT, Chen CM, Li CH (2011). Early endoscope-assisted hematoma evacuation in patients with supratentorial intracerebral hemorrhage: case selection, surgical technique, and long-term results. Neurosurg Focus.

[26] Li Y, Wang J, Li Z (2018). Computed tomography angiography spot sign as an indicator for ultra-early stereotactic aspiration of intracerebral hemorrhage. World Neurosurg.

[27] Li Y, Yang S, Zhou X, Lai R, Tan D (2022). A retrospective cohort study of neuroendoscopic surgery versus traditional craniotomy on surgical success rate, postoperative complications, and prognosis in patients with acute intracerebral hemorrhage. Comput Intell Neurosci.

[28] Ma L, Hou Y, Zhu R, Chen X (2017). Endoscopic evacuation of basal ganglia hematoma: surgical technique, outcome, and learning curve. World Neurosurg.

[29] McCulloch P, Altman DG, Campbell WB (2009). No surgical innovation without evaluation: the IDEAL recommendations. Lancet.

[30] Mendelow AD, Gregson BA, Fernandes HM (2005). Early surgery versus initial conservative treatment in patients with spontaneous supratentorial intracerebral haematomas in the International Surgical Trial in Intracerebral Haemorrhage (STICH): a randomised trial. Lancet..

[31] Mendelow AD, Gregson BA, Rowan EN (2013). Early surgery versus initial conservative treatment in patients with spontaneous supratentorial lobar intracerebral haematomas (STICH II): a randomised trial. Lancet..

[32] Miki K, Yagi K, Nonaka M et al (2018a) Spot sign as a predictor of rebleeding after endoscopic surgery for intracerebral hemorrhage. J Neurosurg:1–6 10.3171/2017.12.JNS172335 29799345

[33] Miki K, Yagi K, Nonaka M (2018). Intraoperative active bleeding in endoscopic surgery for spontaneous intracerebral hemorrhage is predicted by the spot sign. World Neurosurg.

[34] Morgenstern LB, Demchuk AM, Kim DH, Frankowski RF, Grotta JC (2001). Rebleeding leads to poor outcome in ultra-early craniotomy for intracerebral hemorrhage. Neurology..

[35] Mould WA, Carhuapoma JR, Muschelli J (2013). Minimally invasive surgery plus recombinant tissue-type plasminogen activator for intracerebral hemorrhage evacuation decreases perihematomal edema. Stroke..

[36] Pan J, Chartrain AG, Scaggiante J (2020). A compendium of modern minimally invasive intracerebral hemorrhage evacuation techniques. Oper Neurosurg.

[37] Pantazis G, Tsitsopoulos P, Mihas C, Katsiva V, Stavrianos V, Zymaris S (2006). Early surgical treatment vs conservative management for spontaneous supratentorial intracerebral hematomas: a prospective randomized study. Surg Neurol.

[38] Poon MT, Fonville AF, Al-Shahi SR (2014). Long-term prognosis after intracerebral haemorrhage: systematic review and meta-analysis. J Neurol Neurosurg Psychiatry.

[39] Rabinstein AA, Anderson CD (2015). Time is brain also counts for ICH. Neurology..

[40] Rodriguez-Luna D, Coscojuela P, Rubiera M (2016). Ultraearly hematoma growth in active intracerebral hemorrhage. Neurology..

[41] Rothrock RJ, Chartrain AG, Scaggiante J (2021). Advanced techniques for endoscopic intracerebral hemorrhage evacuation: a technical report with case examples. Oper Neurosurg.

[42] Ruiz-Sandoval JL, Chiquete E, Romero-Vargas S, Padilla-Martinez JJ, Gonzalez-Cornejo S (2007). Grading scale for prediction of outcome in primary intracerebral hemorrhages. Stroke..

[43] Scaggiante J, Zhang X, Mocco J, Kellner CP (2018). Minimally invasive surgery for intracerebral hemorrhage. Stroke..

[44] Sondag L, Schreuder F, Boogaarts HD (2020). Neurosurgical intervention for supratentorial intracerebral hemorrhage. Ann Neurol.

[45] Song R, Ali M, Smith C (2022). Initial experience with the NICO myriad device for minimally invasive endoscopic evacuation of intracerebral hemorrhage. Oper Neurosurg.

[46] Spiotta AM, Fiorella D, Vargas J (2015). Initial multicenter technical experience with the Apollo device for minimally invasive intracerebral hematoma evacuation. Neurosurgery..

[47] Sun GC, Chen XL, Hou YZ (2017). Image-guided endoscopic surgery for spontaneous supratentorial intracerebral hematoma. J Neurosurg.

[48] van Asch CJ, Luitse MJ, Rinkel GJ, van der Tweel I, Algra A, Klijn CJ (2010). Incidence, case fatality, and functional outcome of intracerebral haemorrhage over time, according to age, sex, and ethnic origin: a systematic review and meta-analysis. Lancet Neurol.

[49] van Asch CJ, Velthuis BK, Rinkel GJ (2015). Diagnostic yield and accuracy of CT angiography, MR angiography, and digital subtraction angiography for detection of macrovascular causes of intracerebral haemorrhage: prospective, multicentre cohort study. BMJ..

[50] Vespa P, Hanley D, Betz J (2016). ICES (intraoperative stereotactic computed tomography-guided endoscopic surgery) for brain hemorrhage: a multicenter randomized controlled trial. Stroke..

[51] Volbers B, Willfarth W, Kuramatsu JB (2016). Impact of perihemorrhagic edema on short-term outcome after intracerebral hemorrhage. Neurocrit Care.

[52] Xia Z, Wu X, Li J (2018). Minimally invasive surgery is superior to conventional craniotomy in patients with spontaneous supratentorial intracerebral hemorrhage: a systematic review and meta-analysis. World Neurosurg.

[53] Ye Y, Wang Q, Ou W, He J, Zhao Z (2020). Endoscopic surgery without decompressive craniectomy in large putaminal intracerebral hemorrhage: assessment of efficacy and safety. Neurocrit Care.

[54] Yushkevich PA, Piven J, Hazlett HC (2006). User-guided 3D active contour segmentation of anatomical structures: significantly improved efficiency and reliability. Neuroimage..

[55] Zhou X, Xie L, Altinel Y, Qiao N (2020). Assessment of evidence regarding minimally invasive surgery vs. conservative treatment on intracerebral hemorrhage: a trial sequential analysis of randomized controlled trials. Front Neurol.

