# Supplementary material for: Safety and technical efficacy of early minimally invasive endoscopy-guided surgery for intracerebral haemorrhage: the Dutch Intracerebral haemorrhage Surgery Trial pilot study
Source: Acta Neurochir (Wien). 2023 Apr 27;165(6):1585–96. doi: 10.1007/s00701-023-05599-2 (PMC10134719; doi:10.1007/s00701-023-05599-2)

**Online resource**

Table of contents

1. Online resource table 1: Group authors with affiliations
2. Online resource data: Surgical protocol.
3. Online resource table 2: Baseline characteristics and technical outcome parameters according to baseline ICH volume subgroups.
4. Online resource table 3: Description of patients with a primary safety outcome and causes of death.
5. Online resource table 4: Mortality and predicted mortality based on ICH-GS at 30 days.
6. Online resource figure: Percentage ICH volume reduction in the first half of patients versus the second half of patients.

Online resource table 1: Group authors and affiliations

| **First name** | **Middle initials** | **Last name** | **Degree** | **Affiliation** |
| --- | --- | --- | --- | --- |
| Ale |  | Algra | MD, PhD | Utrecht Stroke Center, Department of Neurology and Neurosurgery and Julius Center University Medical Center Utrecht, The Netherlands. |
| Craig |  | Anderson | MD, PhD, FRACP | The George Institute for Global Health  *and* Faculty of Medicine, UNSW, Sydney, Australia |
| Jelis |  | Boiten | MD, PhD | Department of Neurology, Haaglanden Medical Center, The Hague, the Netherlands |
| Hieronymus | D. | Boogaarts | MD, PhD | Department of Neurosurgery, Radboud University Medical Center, Nijmegen, The Netherlands |
| Paul | J.A.M. | Brouwers | MD, PhD | Department of Neurology, Medisch Spectrum Twente, Enschede, the Netherlands |
| Anil |  | Can | MD | Amsterdam UMC, University of Amsterdam, Department of Neurosurgery,  *and* Amsterdam Neurosciences, Neurovascular Disorders, Meibergdreef 9, Amsterdam, The Netherlands |
| Jonathan |  | Coutinho | MD, PhD | Department of Neurology, Amsterdam University Medical Centers, Academic Medical Center, Amsterdam, the Netherlands |
| Ruben |  | Dammers | MD, PhD | Department of Neurosurgery, Erasmus MC University Medical Center, Rotterdam, the Netherlands |
| Diederik | W.J. | Dippel | MD, PhD | Department of Neurology, Erasmus MC University Medical Center, Rotterdam, the Netherlands |
| M. | Heleen | den Hertog | MD, PhD | Department of Neurology, Isala Hospital, Zwolle, the Netherlands |
| Dana |  | Holl | MD | Department of Neurosurgery, Erasmus MC University Medical Center, Rotterdam, the Netherlands |
| Paula | M. | Janssen | MD | Department of Neurology, Erasmus MC University Medical Center, Rotterdam, the Netherlands |
| Wilmar | M.T. | Jolink | MD, PhD | Department of Neurology, Isala Hospital, Zwolle, the Netherlands |
| L. | Jaap | Kappelle | MD, PhD | Department of Neurology, Brain Center Rudolf Magnus, University Medical Center Utrecht, Utrecht, the Netherlands |
| Kuan | H. | Kho | MD | Department of Neurosurgery, Medisch Spectrum Twente, Enschede, the Netherlands; Clinical Neurophysiology Group, University of Twente, Enschede, the Netherlands |
| Catharina | J.M. | Klijn | MD. PhD | Department of Neurology, Donders Institute for Brain, Cognition and Behaviour, Radboud University Medical Center, Nijmegen, The Netherlands |
| Radboud | W. | Koot | MD, PhD | Department of Neurosurgery, Leiden University Medical Center, Leiden, the Netherlands |
| Paul | L.M. | de Kort | MD, PhD | Department of Neurology, Elisabeth-Twee Steden Ziekenhuis, Tilburg, the Netherlands |
| Wouter | A. | Moojen | MD, PhD | Department of Neurosurgery, Haaglanden Medical Center, The Hague, the Netherlands |
| Dharmin |  | Nanda | MD, PhD | Department of Neurosurgery, Isala Hospital, Zwolle, the Netherlands |
| Sjoert | A.H. | Pegge | MD | Department of Medical Imaging, Radboud University Medical Center Nijmegen, The Netherlands |
| Wilco | C. | Peul | MD, PhD, MBA | Leiden University Department of Neurosurgery, The Hague & Leiden, the Netherlands. |
| Bram |  | van der Pol | MD, PhD | Department of Neurosurgery, Elisabeth-Twee Steden Ziekenhuis, Tilburg, the Netherlands |
| Inger | R | de Ridder | MD, PhD | Department of Neurology, Cardiovascular Research Institute Maastricht, Maastricht University Medical Center, Maastricht, the Netherlands |
| Floris | H.B.M. | Schreuder | MD, PhD | Department of Neurology, Donders Institute for Brain, Cognition and Behaviour, Radboud University Medical Center, Nijmegen, The Netherlands |
| Lotte |  | Sondag | MD | Department of Neurology, Donders Institute for Brain, Cognition and Behaviour, Radboud University Medical Center, Nijmegen, The Netherlands |
| Onno | P.M. | Teernstra | MD, PhD | Department of Neurosurgery, Maastricht University Medical Center, Maastricht, the Netherlands |
| W. | Peter | Vandertop | MD, PhD | Amsterdam UMC, University of Amsterdam, Department of Neurosurgery, Amsterdam Neurosciences, Neurovascular Disorders, Meibergdreef 9, Amsterdam, The Netherlands  AND  Amsterdam UMC, Vrije Universiteit Amsterdam, Department of Neurosurgery, Amsterdam Neurosciences, Neurovascular Disorders, De Boelelaan 1117, Amsterdam, Netherlands |
| Marieke | J.H. | Wermer | MD, PhD | Department of Neurology, Leiden University Medical Center, Leiden, the Netherlands |
| Albert |  | van der Zwan | MD, PhD | Department of Neurosurgery, Brain Center Rudolf Magnus, University Medical Center Utrecht, Utrecht, the Netherlands |

Online resource data: Surgical protocol

NL63100.078.17 / Surgical protocol accompanying the Dutch ICH Surgery Trial pilot study; minimally-invasive endoscopy-guided surgery for spontaneous ICH

Version 3.0 November 1^st^, 2019

Introduction

This surgical protocol is a supplement to the “RESEARCH PROTOCOL the Dutch ICH Surgery Trial pilot study; minimally-invasive endoscopy-guided surgery for spontaneous ICH”. This study intends to study the safety, feasibility, and technical efficacy of minimally-invasive endoscopy-guided surgery for the treatment of supratentorial intracerebral hematoma (ICH). Moreover, the potential effect on functional outcome will be estimated through this multicentre, prospective, intervention study.

To ensure minimal performance bias we outline a surgical protocol to which including centres are obliged to adhere.

For details on study population, patient eligibility, and study procedures we refer to the RESEARCH PROTOCOL Sections 4 “Study Population” and 8 “Methods”.

Investigational product

The investigational product is a device for minimally-invasive, endoscopy-guided hematoma removal. For this study Artemis^TM^ Neuro Evacuation Device system will be used. For more details on the investigational product itself and a review of its use to date be referred to the RESEARCH PROTOCOL Section 6 “Investigational Product”.

The choice of any particular device is left to the discretion of the neurosurgeon. When other devices will become available, they may be used when they are deemed admissible by the steering committee.

Surgical protocol

All patients included in the study receiving minimally-invasive endoscopy-guided surgery will be treated according to this surgical protocol and according to local institutional guidelines.

Training

Surgeons will undergo a detailed instructional training on the stereotactic-guided endoscopic procedure including direct mentoring of the detailed step-by-step surgical protocol by the surgical principal investigator and hands-on training in a dry-lab setting. The latter will be provided by Penumbra, manufacturer of the Artemis^TM^ Neuro Evacuation Device systems at the IRCAD training facility in Strasbourg, France, or a similar set-up at another location.

Pre-operative neuroimaging and planning for frameless image-guided endoscopic surgery

Depending on the institution and neuronavigation systems used, appropriately protocolled CT-imaging studies will be uploaded into the neuronavigation software for procedural planning and guidance. The use of surface merging or fiducial markers will be at the discretion of the surgeon. The timing of the neuronavigation (non-contrast CT) scan will be as soon as possible after informed consent. A trajectory will be selected that is both technically feasible and allows access to the longest possible axis of the hematoma. For this, we adhere to the protocol as described in the ICES study.^38^ The ideal trajectory, which is parallel to the long axis of the hematoma, is selected determining a candidate entry and target point. One of three approaches will be selected: (A) anterior frontal lobe approach, (B) posterior parietal lobe approach, (C) surface cortical approach, each of which will be designed to be parallel and in the middle of the long axis of the hematoma while avoiding the internal capsule, vasculature, eloquent white matter tracts, and ventricles.

Surgical procedure

Patients will be placed upon the procedural table according to the approach used. The procedures will be performed under general anaesthesia and prophylactic antibiotics will be administered according to local protocol. An external localization array or other neuronavigation localization will be placed for registration according to the neuronavigation system in use. Once the appropriate entry point is identified, this area will be prepared and sterile draped according to institutional guidelines.

The image-guidance probe is positioned over the candidate entry point. The virtual extension of the probe tip can be employed to interrogate the candidate entry points to assess whether or not the endoscope sheath will transgress any critical functional areas. If need be, the entry point can be adapted intra-operatively.

A 1.5-2.0 cm burr hole or minicraniotomy will then be created in a standard manner of a size large enough to accommodate the selected endoscopy sheath. The dura is opened and the cortical surface coagulated and incised. A localization array (e.g., Instrument Adapter Clamp with Instrument Adapter Array, Brainlab AG) will be attached to the selected neuroendoscopic sheath and registered to the navigation system.

The sheath will then be advanced using neuronavigation into the targeted landing zone within the distal aspect, typically at 2/3 of the way along the long axis, of the hematoma (point # 1) and the inner obturator removed. The sheath will then be stabilised (e.g., manually stabilised, mechanically stabilised, or peeled away and stapled down) into position. The neuroendoscope will then be inserted into the sheath and under direct visualization the Artemis^TM^ Neuro Evacuation Device will be placed through the working channel of the trocar. The sheath will be irrigated at the discretion of the operator using the irrigation port of the endoscope and the irrigant will be intermittently aspirated with the Artemis^TM^ system until a clear working view is created within the sheath that allows visualization of the surgical field at the sheath tip. The Artemis^TM^ wand will be advanced under direct visualization to, or just beyond the tip of the sheath and actuated to evacuate the blood products. If the working view becomes obscured by blood products within the sheath, additional irrigation and aspiration will be performed intermittently to clear the field. This will be repeated until no further clot can be evacuated at this location. The endoscope sheath is then irrigated to be sure that there is no evidence of active bleeding. If active bleeding is detected, then irrigation will be continued until the bleeding stops. If the bleeding will not stop adequately, the endoscope will be introduced into the sheath, fixed in place, and the bleeding point identified endoscopically. Once haemostasis is obtained the endoscope sheath is retracted to a point approximately 1/3 of the way into the hematoma cavity (point # 2). The suctioning and irrigation process is then repeated at point # 2. Suctioning will continue until at least 75-80% of the hematoma volume is thought to be removed. Lastly, the endoscope is introduced to be sure there is no sign of any ongoing streams of blood coming from any vessels, which might require coagulation. However, no rotational steering of the sheath or lateral exploration of the hematoma cavity, will be permitted. The cortical surface is inspected carefully to be sure that there is no ongoing bleeding from the corticotomy. The dura and skin are closed in a routine manner. A control NCCT is performed immediately after surgery or when possible intra-operatively (hybrid room, intra-operative CT) to confirm adequate hematoma evacuation and to assess for any complications (e.g., rebleeding, hydrocephalus, increased mass effect). The surgical goal is to achieve at least a 75-80% reduction in haemorrhage volume and/or a residual volume of <15mL. It is at the surgeon’s discretion to opt to immediately return to the OR to evacuate any residual hematoma.

Postoperative care protocol

Patients are either admitted to the (neuro-)ICU or a dedicated Stroke Unit for postoperative care. Neurological evaluation will be performed as per institutional guidelines. Hypertension will be treated according to National Guidelines as will be the standard treatment for patients not in the study’s surgical arm. For this, a systolic blood pressure of no higher than 160 mmHg will be strived for.

Ideally, patients should emerge rapidly from anaesthesia to permit immediate assessment of the results of surgery and to provide a baseline for continuing postoperative neurologic follow-up. Nevertheless, there are some categories of patients in whom early awakening will not be deemed appropriate by the attending neurosurgeon (e.g., preoperative obtunded consciousness or inadequate airway control, high postoperative risk of brain oedema, raised ICP, or deranged intracerebral haemostasis). This will remain at the discretion of the surgeon.

Prophylactic use of low-molecular-weight heparin (LMWH) in immobile patients will be allowed. Intermittent pneumatic compression and elastic stockings can be applied in the first 72 hours,

Restarting anticoagulant or antiplatelet therapy in patients with a clear indication will be allowed as of three days after surgery. There are, however, no trials to aid in this decision-making.

Online resource table 2: Baseline characteristic and technical outcome parameters according to baseline ICH volume subgroups.

|  | Baseline ICH volume <30mL (n=11)* | Baseline ICH volume ≥ 30mL (n=29) |
| --- | --- | --- |
| Age, mean (SD) | 56 (15) | 61 (13) |
| Male sex, n (%) | 7 (64) | 21 (72) |
| Glasgow coma scale score, median (IQR) | 14 (10-15) | 11 (9-13) |
| NIHSS score, median (IQR) | 18 (13-20) | 20 (15.5-23.5) |
| ICH volume baseline, median (IQR) | 24.5 (18.1-28.5) | 60.3 (42.6-75.5) |
| Intraventricular extension, n (%) | 5 (46) | 14 (48) |
| ICH-GS score at inclusion, median (IQR) | 7 (6-7) | 9 (8-9) |
| Time from symptom onset to surgery (first cut) (hours, min), median (IQR) | 6h50min (4h54min-8h13min) | 6h35min (5h30min-7h49min) |
| Duration of procedure from first incision to skin closure (min), median (IQR) | 63 min (52 min-96 min) | 76 min (56 min-111 min) |
| Active bleeding present during surgery, n (%) | 8 (73) | 11 (38) |
| ICH volume at 24 hours, median (IQR) | 3.4 (2.0-22.3) | 12.3 (6.3-26.8) |
| Absolute ICH volume reduction, median (IQR) | 13.1 (0.7-19.6) | 39.9 (28.0-52.7) |
| Percentage ICH volume reduction, median (IQR) | 80.7 (3.9-91.4) | 77.6 (52.3-87.7) |
| Patients with clot volume reduction ≥60%, n (%) | 7 (64) | 21 (72) |
| Patients with clot volume reduction ≥80%, n (%) | 6 (55) | 13 (45) |
| Patients with remaining clot volume ≤15mL, n (%) | 7 (64) | 16 (55) |
| Conversion to craniotomy, n (%) | 0 (0) | 1 (3) |
| Death within 30 days | 0 (0) | 4 (14) |

*Only 2 patients with baseline ICH volume <15mL with both a ICH volume reduction of >80%.

Online resource table 3: Description of patients with a primary safety outcome and causes of death

| **Description of SAE that led to the primary outcome** | **Primary outcome** | **Clinical outcome at 7 days** |
| --- | --- | --- |
| 82-year-old patient, baseline GCS E4M6V1, NIHSS 22, ICH volume 90.8mL, with IVH, ICH-GS 11. Patient died because of neurological deterioration due to ICH haemorrhage progression despite surgery. Surgical procedure was stopped. Came from another hospital; on repeated CT repeated upon arrival in neurosurgical centre progression of ICH with large spot sign and clinical deterioration. | Death ≤24 hours | Death ≤24 hours |
| 63-year-old patient, baseline GCS E3M6V4, NIHSS 18, ICH volume 24.5mL, without IVH, ICH-GS 6. Patient deteriorated in the emergency room before going for surgery (to E2M5V2), no new imaging was performed. No new NIHSS score was performed before surgery. Presumed cause was intracerebral haemorrhage progression. At 24 hours NIHSS was 22. | NIHSS increase ≥4 points at 24 hours | NIHSS 12 |
| 59-year-old patient, baseline GCS E1M5V1, NIHSS 25, ICH volume 79.2mL, with extensive IVH, ICH-GS 11. On clinical examination at 24 hours, patient was intubated and had an NIHSS score of 29. | NIHSS increase ≥4 points at 24 hours | NIHSS 28 |
| 58-year-old patient, baseline GCS E3M5V1, NIHSS 20, ICH volume 60.4mL, without IVH, ICH-GS 8. On clinical examination at 24 hours, patient was intubated and therefore had an NIHSS of 30. | NIHSS increase ≥4 points at 24 hours | NIHSS 18 |
| 43-year-old patient, baseline GCS E4M6V5, NIHSS 7, ICH volume 28.1mL, with extensive IVH, ICH-GS 6. Patient deteriorated before surgery to E1M5V2 with progression of ICH and hydrocephalus. No new NIHSS score was performed, and patient was intubated. On examination the patient was intubated and had an NIHSS of 33. | NIHSS increase ≥4 points at 24 hours | NIHSS 27 |
| 71-year-old patient, baseline GCS E3M6V5, NIHSS 13, ICH volume 28.5mL, without IVH, ICH-GS 7. On clinical examination at 24 hours, patient deteriorated to an NIHSS of 18. | NIHSS increase ≥4 points at 24 hours | NIHSS 16 |

| **Description of SAE leading to death < 30 days** | **Interval from ictus to SAE (days)** | **Interval from ictus to death (days)** |
| --- | --- | --- |
| 82-year-old patient, baseline GCS E4M6V1, NIHSS 22, ICH volume 90.8mL, with IVH, ICH-GS 11. (This patient was the same patients as a primary outcome patient, descripted in the table above that died within 24 hours)  Patient died because of neurological deterioration due to the intracerebral haemorrhage progression, progression of bleeding despite surgery. Surgical procedure was stopped. Came from another hospital; on repeated CT repeated upon arrival in neurosurgical centre progression of ICH with large spot sign and clinical deterioration. | 0 | 0 |
| 57-year-old patient, baseline GCS E3M6V1, NIHSS 17, ICH volume 55.8mL, with IVH, ICH-GS 9.  After the diagnosis of pulmonary embolism on day 11 after surgery, therapeutic heparin was started. On day 12 clinical deterioration to EMV 1-1-1 with dilated wide pupils, based on a new haemorrhage at the site of the initial haemorrhage for which the patient was operated. Treatment by external ventricular drains (opening pressure of 50cmH20), after which patients pupils became less wide and possibly reactive to light. Patient died after a palliative care decision was made. | 11 | 12 |
| 82-year-old patient, baseline GCS E1M5V1, NIHSS 32, ICH volume 34.5mL, with IVH, ICH-GS 10.  Patient was discharged from ICU at neurosurgical centre to ICU of general hospital, still intubated. After 1 week in this hospital, the family and treating physicians no longer wanted the patient to be dependent of the tube, and the patient was extubated and palliative care was started. He died a week later. | 16 | 21 |
| 81-year-old patient, baseline GCS E4M5V1, NIHSS 25, ICH volume 76.9mL, without IVH, ICH-GS 10.  Presumably progression of haemorrhage or oedema (no additional imaging was performed), refrained from further treatment, went to hospice and died there. | 22 | 22 |

*CT, computed tomography scan; GCS, Glasgow coma scale score; ICH, intracerebral haemorrhage; ICH-GS, intracerebral haemorrhage grading scale; ICU, intensive care unit; IVH, intraventricular haemorrhage (extension); NIHSS, National Institutes of Health Stroke Scale; SAE, serious adverse event*

Online resource table 4:
Expected and observed deaths at 30 days according to ICH-GS scores

| **ICH-GS** | **Expected death at 30 days, %** | **DIST pilot study, n** | **DIST pilot study expected number of deaths at 30 days, n** | **DIST pilot study observed deaths at 30 days, n (%)** | **Death ratio**  **observed/expected** |
| --- | --- | --- | --- | --- | --- |
| 5 | 17% | 1 | 0.2 | 0 | 0 |
| 6 | 8% | 3 | 0.2 | 0 | 0 |
| 7 | 20% | 9 | 1.8 | 0 | 0 |
| 8 | 43% | 10 | 4.3 | 0 | 0 |
| 9 | 71% | 11 | 7.8 | 1 | 0.1 |
| 10 | 87% | 4 | 3.5 | 2 | 0.6 |
| 11 | 100% | 2 | 2 | 1 | 0.5 |
| Total |  | 40 | 19.8 | 4 | 0.2 |

*ICH-GS, intracerebral haemorrhage grading scale*

Online resource figure


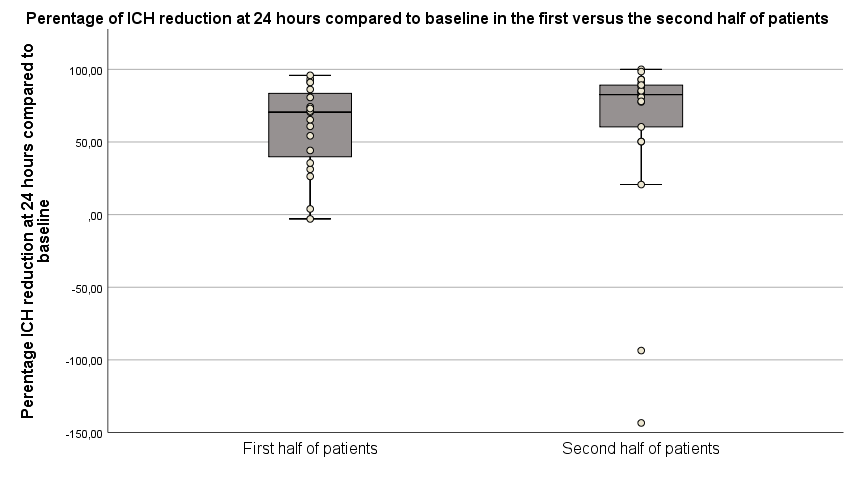

Supplement: Supplementary file 1 — (DOCX 59 kb) [file 701_2023_5599_MOESM1_ESM.docx]
